# Heart Rhythm Analyzed via Shapelets Distinguishes Sleep From Awake

**DOI:** 10.3389/fphys.2019.01554

**Published:** 2020-01-17

**Authors:** Albert Zorko, Matthias Frühwirth, Nandu Goswami, Maximilian Moser, Zoran Levnajić

**Affiliations:** ^1^Complex Systems and Data Science Lab, Faculty of Information Studies in Novo Mesto, Novo Mesto, Slovenia; ^2^Human Research Institute, Weiz, Austria; ^3^Physiology Division, Otto Loewi Research Center of Vascular Biology, Immunity and Inflammation, Medical University of Graz, Graz, Austria

**Keywords:** respiratory sinus arrhythmia, time series analyses, shapelets, onset of sleep, heart rate variability, logRSA

## Abstract

Automatically determining when a person falls asleep from easily available vital signals is important, not just for medical applications but also for practical ones, such as traffic safety or smart homes. Heart dynamics and respiration cycle couple differently during sleep and awake. Specifically, respiratory modulation of heart rhythm or *respiratory sinus arrhythmia* (RSA) is more prominent during sleep, as both sleep and RSA are connected to strong vagal activity. The onset of sleep can be recognized or even predicted as the increase of cardio-respiratory coupling. Here, we employ this empirical fact to design a method for detecting the change of consciousness status (sleep/awake) based only on heart rate variability (HRV) data. Our method relies on quantifying the (self)similarity among *shapelets* – short chunks of HRV time series – whose “shapes” are related to the respiration cycle. To test our method, we examine the HRV data of 75 healthy individuals recorded with microsecond precision. We find distinctive patterns stable across age and sex, that are not only indicative of sleep and awake, but allow to pinpoint the change from awake to sleep almost immediately. More systematic analysis along these lines could lead to a reliable prediction of sleep.

## Introduction

Determining the status of consciousness (being awake or asleep) is usually done in a sleep lab by sophisticated polygraphic recordings (Quintana-Gallego et al., 2004). Under real life conditions it would be preferable to do this online from automated analysis of vital signs recorded with minimally obtrusive sensor systems. In fact, several approaches have already been explored (Canisius and Penzel, 2007; Romine et al., 2019; Sadek et al., 2019) to achieve this. Various medical applications are easy to imagine, but perhaps more important are practical applications in which vigilance plays an important role. An obvious example is transportation, where the driver of a bus, train or a plane must stay awake at all times. Such an algorithm could be also translated into an alarm system that activates when the algorithm ‘recognizes’ that the driver is at risk of falling asleep. Statistics show that a significant number of car accidents were probably due to driver falling asleep (Horne and Reyner, 1995; Royal, 2003; Ftouni et al., 2012).

Any algorithm that automatically determines whether a person is awake or asleep (awake status) needs constant access to the body’s vital signals. Those signals (data) should be processed continuously, so that patterns in the data that indicate sleep (or reduced vigilance) could be spotted immediately. Of course, the practical interest is to detect the change in consciousness status as soon as possible. Even more useful would be to *predict* the onset of sleep, so that the alarm can be triggered in a timely manner. In such a setting, false positives (alarm goes off, but the driver is awake) are far less dangerous than false negatives (the opposite).

The precision in determining consciousness status involves two aspects: (*i*) correctly recognizing the onset of sleep or reduction of vigilance and minimize the rate of false positives (accuracy), and (*ii*) recognizing the onset as quickly as possible, or even better, predicting it. Both depend on the quality of available data as well as the sophistication of data analysis. A myriad of new data analysis approaches rose in the last decades in response to increased availability and richness of datasets in varying domains of society, science, and technology. Modern methods of time series analysis are able to identify, quantify and compare virtually any pattern of interest with great accuracy and even from noisy data (Richman and Moorman, 2000; Yaffee and McGee, 2000; Bevington and Robinson, 2003; Small, 2005; Bendat and Piersol, 2010; Grote et al., 2019; Zou et al., 2019).

Besides data analysis, quality of this determination will also depend on whether any prior data of vital signs for that person are available. Ideally, an algorithm should establish the status of consciousness without prior data from the same person, which is a very challenging task given that body processes related to falling asleep differ from person to person considerably (Ogilvie, 2001). In contrast, with the prior data available, the awake status will be identifiable faster and with better precision. Another factor is the presence of noise and incompleteness in the vital sign data: fortunately, these can be significantly reduced thanks to modern measuring equipment.

Which vital signal or signals are most useful for such an algorithm? The best choices to measure vigilance are vital signals that are easy to measure with good precision and signals that change in synchrony with the sleep and awake states or at least indicate a transition between these states. One such signal is the phase and frequency coordination (synchrony) between respiratory and heart rhythm known as *respiratory sinus arrhythmia* (RSA), which is observed in the sequence of time intervals between consecutive heart beats (Moser et al., 1994, 1995; Yasuma and Hayano, 2004; Bartsch et al., 2005; Denwer et al., 2007). Modern Holter (or similar) devices can measure RSA with microsecond precision, which more than suffices for application to the problem considered here (Lynn et al., 2013; Barrett et al., 2014; American Heart Association, 2015). Measuring RSA can be done with minimal hinderance of the person’s normal activities by belt or glue electrodes from a unipolar ECG taken from the chest or hands. There are also other forms of coupling related to cardio-respiratory phase synchronization and cardio-respiratory time delay stability (Bartsch et al., 2014), but these involve the high resolution and synchronized recordings of respiration, which is usually not available in clinical settings. This suggests that RSA is a more suitable choice of vital data for our study, where we choose not to quantify vagal activity via RSA, but to investigate the similarity of sequences of HRV data carrying different amounts of RSA information.

**FIGURE 1:**
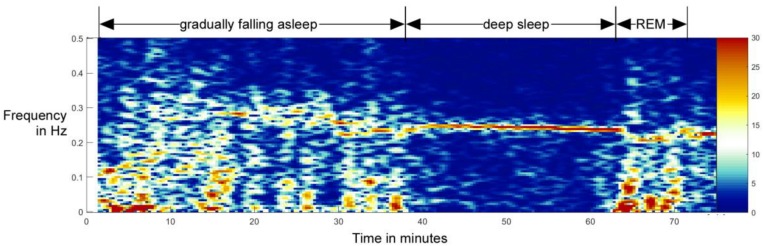
Example of the transition from awake state to non-REM sleep and later to REM sleep in a 10-year-old boy. In this spectral analysis of HRV, one can clearly see the gradual formation and increase of RSA as a band around 0.25 Hz. Although the total activity is higher during awake state, the activity in the RSA band stands out during the non-REM sleep – falling asleep qualitatively resembles a phase transition normally studied in physics (Bartsch et al., 2012; Penzel et al., 2016). This process is reversed when entering the REM stage (from minute 63 on). This figure is not a part of the study reported in this paper, but it was done independently, as part of another study reported in Bonin et al. (2004).

But how to precisely define sleeping vs. awake from RSA data? The gold standard for determination of being asleep including sleep staging is polygraphy, including EEG, ECG, EMG, respiration, and movement sensors (Kaplan et al., 2017; National Institute of Neurological Disorders and Stroke, 2019). On the other hand, first bodily signs of sleep are shown at the autonomic level, as brainstem activity controls the sleep stages and the brain centers for respiration and circulation are anatomically close to sleep-induction centers. Additionally, heart and respiratory cycle are coupled differently during the sleep and awake states (Moser et al., 2006). While falling asleep, the heart rhythm gets gradually more modulated by respiration, which indicates increasing vagal control of the heart (Chouchou and Desseilles, 2014; Niizeki and Saitoh, 2018). This is illustrated in a sleep onset recording done on a 10-year-old boy (Figure 1), measured before and after falling asleep.

During deep sleep, heart and respiratory oscillations are maximally coupled to one another, which corresponds to maximal RSA and is a reliable indicator of autonomic regulation of sleep. RSA indicates the presence of strong vagal oscillations synchronous to respiration, which regulates (speeds up or slows down) the heart rhythm (Moser et al., 1994). The increase of cardio-respiratory coupling (the increase of order of RSA) is hence the first sign that the body is falling asleep. For the purpose of this work, we identify the onset of sleep with the onset of RSA. This onset is detectable from heart rate variability (HRV) data, which is the main topic of this paper.

However, identifying the onset of RSA from HRV data alone is challenging and requires a good choice of data analysis methods. In contrast to previous studies (Billman, 2011; Billman et al., 2015), rather than considering time and frequency domain parameters of RSA, we employ shapelet analysis, which has several advantages and was revealed as useful in analyzing biomedical data (Xi et al., 2006; Ye and Keogh, 2009; Hills et al., 2013, 2014; Rakthanmanon and Keogh, 2013). Shapelets are short segments of HRV time series that are repetitively compared to prior and past parts of the original time series. Their self-similarity and pairwise distances can be precisely classified, including the similarity to any pre-selected shapelet. Using this framework, we identify the shapelet whose distances to all other time intervals generates the best distinction between sleep and awake. We examine how this self-similarity changes as the subject transits from awake to sleep, which allows us to pinpoint the onset of sleep (change of consciousness status) with good precision.

The aim of this paper is to propose a new methodology for analyzing awake-to-sleep transition and discuss its merits for a practical and useful algorithm. We construct a shapelets-based method relying on standard approaches in non-linear time series analysis. Then, using the statistics of shapelet comparison, we define a similarity threshold that we show is a reliable indicator of the change of awake status. As an intermediate step in our analysis we identify the *best shapelet* – the most self-similar shapelet in the HRV time series – and show that its length is comparable to the multiple length of a typical respiration cycle. This confirms that HRV time series at the onset of sleep are most self-similar at the RSA time-scale, as expected from the definition of RSA.

To test our methods, we use the data from 75 healthy individuals of varying age and sex whose circadian (diurnal) 24 h HRV data were recorded with microsecond precision. Our data also include the self-reported information about when the subject fell asleep and when he/she woke up. This narrows our search since we look for the onset of RSA close to the time when subject declared going to sleep. We show that our method can pinpoint the change of awake status with a good precision using only HRV data – but of course, only as long as a representative sample of a person’s 24 h HRV data was available. As our subject sample is relatively small, we were unable to make any substantial prediction of the onset of sleep. However, we were able to identify that a subject was asleep almost immediately after he/she fell asleep. We found that HRV self-similarity patterns relevant for this identification are fairly stable across age and sex. This suggests that a more systematic analysis with larger and more diverse sample sets could lead to automating this procedure, possibly even without prior data. Along the same lines, this is a step toward an algorithm for early-warning of falling sleep.

## Subjects and Measurements

### Subjects

The data was collected within the setting of workplace-related health assessment. We made 24 h-measurements of HRV from 75 participants, 40 men (age 16–57, mean ± SD: 34.7 ± 11.0) and 35 women (age 16–56, mean ± SD: 37.0 ± 13.5). All subjects declared themselves to be in good general health, and with no prior history of cardiologic problems or other medical conditions that would influence heart or autonomic activity. Each subject agreed to wear a portable Holter monitor for an entire day, while carrying on with his/her routine activities on that day, including sleeping during the night. Subjects had given their written consent to participate in the study beforehand and received feedback on their results after completion. The study protocol complied with the guidelines of “good clinical practice” (ICH-GCP) following the declaration of Helsinki and with the regulations of the National Data Protection Act (Section 14 Abs. 1DSG 2000). Since this study involved only healthy subjects without endangering their health, and since it involved no medical diagnoses, interventions or treatments, according to the local legislation in Austria the study did not require an approval from the University’s ethics committee.

### Heart Rate Variability Measurements and Data

We used a single-channel high-precision ECG monitor (ChronoCord^1^, 7th generation, Joysys, Weiz, Austria, sample rate: 8000 Hz, resolution: 16 bit) (Joysys, 2018) to continuously record intervals between heartbeats (Noble et al., 1990; Gallasch et al., 1996, 1997; Pinnell et al., 2007; Surawicz and Knilans, 2008). For the continuous measurements, three adhesive electrodes were applied on the trunk of the participants (sternum, 5th left intercostal space, and a reference electrode on the right side of the trunk between 11th and 12th rib) (Klabunde, 2012; Medical Training and Simulation LLC, 2017). The device was then attached to a belt or the waistband of the subject. During a 24-h period, the device assessed the intervals between heart beats with precision of several microseconds. Data have been stored on an SD card for further evaluation. The subjects were instructed to note the time of light off in the evening and light on in the morning as a best available proxy for falling asleep. Heart beats were detected from ECG by device during recording. For further analysis, they were expressed as R-wave-to-R-wave (RR) intervals (time intervals between two consecutive R waves of heart beat) (Amani et al., 2011). Smaller (respectively, larger) RR values indicate that heart works faster (respectively, slower; Hurst, 1998; Iaizzo, 2005). After the measurements were completed, we extracted for each person the time series (sequence) of RR values. Each of these 75 time series contained about 110,000 RR values. For easier interpretability and with no loss of generality we converted the data from RR intervals to heart rate, expressed in beats per minute (b/min). To reduce above described errors, we removed RR values that were smaller than the minimum ECG value (40 bmp) or higher than 180 bmp, in accordance with the standard procedures in medical sciences (Perski et al., 1992; Krul et al., 2013; Sarzynski et al., 2013). This resulted in removal of the 0.63% of the data (basically negligible).

### Preliminary Analysis

**FIGURE 2:**
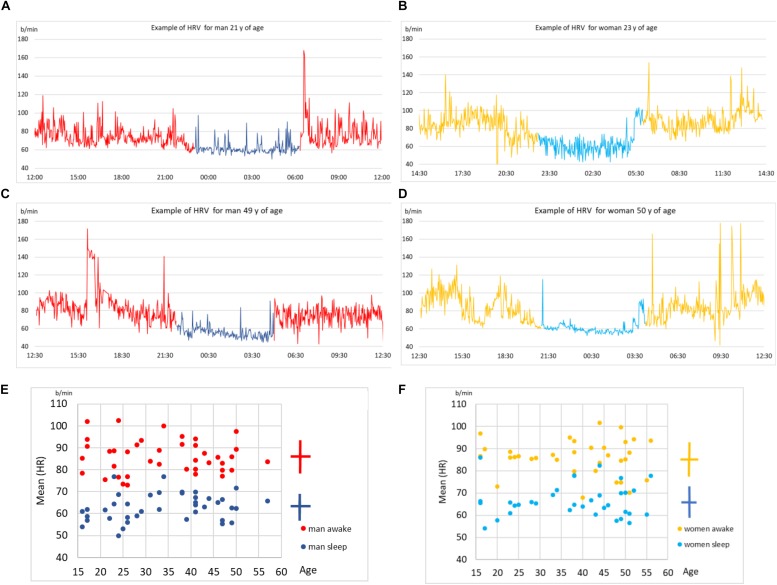
**(A)** Time series of beat-to-beat HRV data (heart rate as function of time) over a day (24 h) for a male subject, 21 years of age. Part of the day when the subject slept is indicated in blue (self-reported information). **(B)** The same data but for a female subject, 23 years of age. **(C)** The same data for another male subject, 49 years of age. **(D)** The same data for another female subject, 50 years of age. **(E)** Scatter plot of age (shown on *x*) vs. mean heart rate (HR, shown on *y*) for all male subjects in our sample. Each male subject is represented as two points, one for sleep mean HR and the other of awake mean HR. Mean value and standard deviation are indicated by the cross symbol next to the scatter plot. **(F)** The same as in **(E)** but for all female subjects in our sample.

We first show some sample results to better illustrate the data. In Figures 2A–D we show four examples of HRV beat-to-beat time series. The values of heart rate (in bpm) are shown as function of time during 24 h of recordings. We show two typical examples for younger subject (male and female, above) and two for middle-aged subjects (below). We indicate in each plot the part of the day when the subject slept (according to self-reported information). In general, the heart beats faster (higher bpm) when a person is awake compared to asleep. While sleeping, heart beat meta-oscillations seem steadier than while awake, especially during non-REM phases. These meta-oscillations indicate autonomic nervous system activity (Moser et al., 1994, 2008) mediated via vagal and sympathetic branches to the sinus node. All plots display quite intense fluctuations during the entire 24 h, which is somewhat more prominent for younger subjects.

In Figures 2E,F we show scatter plots of mean heart rate (HR, shown on y) vs. age (shown on x), for male and female subjects, respectively. Sleep and awake mean HR are calculated separately for each subject (according to self-reported information) and shown by different colors in each scatter plot. Clearly, the heart on average beats slower while asleep. This preliminary analysis shows that while there are qualitative differences in heart activity between sleep and awake, determining the status of consciousness from HRV alone is not sufficient, since none of these simple parameters discriminate it precisely. This stresses the need for more sophisticated data analysis approaches, to which we devote the rest of this paper.

## Shapelet Analysis

In this section we introduce shapelet analysis as our main methodological tool (Xi et al., 2006; Ye and Keogh, 2009; Hills et al., 2013, 2014; Rakthanmanon and Keogh, 2013). In general, our approach belongs to unsupervised learning from data (James et al., 2013; Längkvist et al., 2014; Celebi and Aydin, 2016). We search for the best way to divide a time series in two parts (classes) such that self-similarity of the time series is maximal within each class, and minimal between the classes. In fact, a long-standing challenge in time series classification is how to find the most efficient measure of similarity between two (or more) time series or parts thereof. Many methods in the literature strive to meet these criteria (Kin-Pong and Wai-Chee Fu, 1999; Costa et al., 2005; Liao, 2005; Aboy et al., 2007; Ding et al., 2008; Batista et al., 2011; Yentes et al., 2013), including shapelet analysis, which we chose for its good record in recognizing physical activities from biomedical time-resolved data. In this regard, shapelet analysis is conceptually somewhat similar to wavelet analysis (Daubechies, 1992). Shapelets rely on a simple quantification of similarity/difference between time series, they are fast to compute and provide easily interpretable results with very good accuracy. We developed our own programing codes for the entire analysis that follows without resorting to any specific software.

### What Are Shapelets?

We explain the concept of shapelets by referring directly to our HRV data. We take a time series of RR values (similar analysis could be done with time series of frequencies). We decompose this time series into *segments* (chunks) of 2 min in length (duration). That yields about 700 segments during 24h, depending on the subject. We assume that at least qualitatively, the heart activity does not drastically change within 2 min, i.e., that it is (relatively) stationary during each segment^2^. This is our starting resolution to detect changes of the consciousness status.

Now we consider one 2-min segment and divide it into smaller parts that we call *shapelets*. In other words, a shapelet is a short sub-interval of a 2 min segment and hence of the original 24 h time series. When dividing a segment into shapelets, we do so in three ways:

• Division into two equal halves, each 1 min long (“level 1”),
• Division into four equal quarters, each 30 s long (“level 2”),
• Division into eight equal eighths, each 15 s long (“level 3”).

**FIGURE 3:**
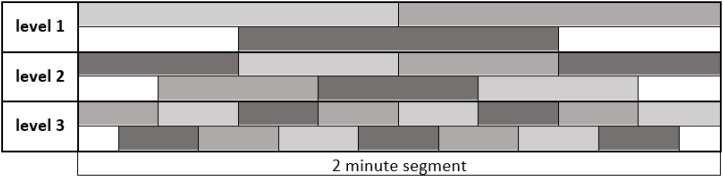
Dividing a 2 min segment of the original 24h time series into shapelets. The length of the entire interval in figure is 2 min, as indicated. Each level contains equally sized adjacent shapelets without overlapping. The original set of shapelets is illustrated as the top sequence at each level. Additional set of shapelets obtained by shifting by half shapelet length is illustrated as bottom sequence at each level. For clarity, shapelets show different shades of gray. In later computations we consider all these shapelets equally, no matter to which level they belong. We call ‘pool of shapelets’ this entire ensemble of shapelets cumulatively.

So, each following level is made of shapelets with half-length of the previous level. At level 1 we obtain 2 shapelets from each segment, each covering a half of the segment, without overlapping. Similarly, at levels 2 and 3 we obtain 4 and 8 shapelets, respectively, jointly covering the entire segment, without overlapping. Besides this main division, at each level we also consider an additional set of shapelets, obtained by shifting the shapelets by half-length at that level. That is to say, at level 1 we obtain one additional shapelet of 1 min length, which is centered at mid-point between the two main shapelets. Similarly, at levels 2 and 3 we obtain 3 and 7 more shapelets, respectively, centered at mid-points between the main set shapelets at each level. We clarify this scheme by illustration in Figure 3, where different shapelets are illustrated by varying tones of gray. We considered additional levels of division, but found them not to contribute to the results: heart activity varies too much on the time scale above 2 min, while below 15 s the resolution becomes too poor. We also examined shapelets down to 1/128 of segment and found no improvement of results.

Choosing the shapelet level defines the resolution of our analysis. For example, at average respiratory rates during sleep, one shapelet of level three will contain about 3–4 respiratory cycles of 4 s duration. Selecting one of the possible resolutions, we can divide all segments of a given time series into shapelets. We can do that also for all levels, obtaining a large ensemble of shapelets, to which we refer as *pool of shapelets*.

### Measuring Distances Between Shapelets

Since we wish to construct a framework for comparing time series (or parts of them, shapelets and segments), we next introduce a distance between a shapelet and a segment. Segments and shapelets can be seen as two time series of different length (duration): a segment is always 2 min long, while shapelet length depends on the level (15 s, 30 s, or 60 s). We first equip ourselves with a measure of similarity between a pair of time series of equal length. We adopt the general Euclidean measure and define the distance *D* between time series *T*_1_ and *T*_2_ as (Faloutsos et al., 1994; Goldin et al., 2004; Deza and Deza, 2009):

$$D\,\left(T_{1},T_{2}\right)=\sqrt{\sum_{i}{\left(x_{i}-y_{i}\right)}^{2}},$$

where *x_i_* are the values belonging to the time series *T*_1_, and *y_i_* to the time series *T*_2_. The sum runs along the index *i* for the entire length of *T*_1_ and *T*_2_. Distance *D* is zero if the time series are identical to one another at each point. In any other case the distance is greater than zero.

**FIGURE 4:**
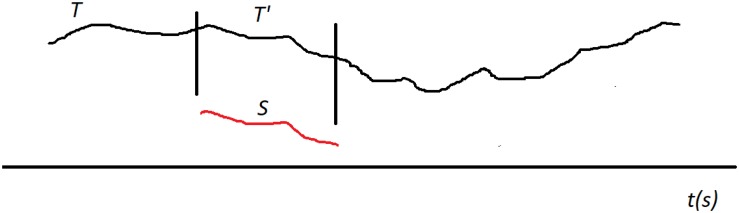
Searching for best matching location between the shapelet *S* (red) and the segment *T* (black). Shapelet is translated (shifted) along *T* from the start to the end, step by step (point by point). At each location we measure the distance *D* between *S* and the corresponding chunk (sub-segment) of the whole segment *T*, which we generically denote with *T*’. The length of *T*’ is always equal to the length of *S*. In this illustration the best matching location of *T*’ is shown, where the distance *D* is minimal. This minimal distance *D* is then taken as the distance δ between *S* and *T*.

Now we generalize this into a distance between a shapelet and a segment called δ. Let us (generically) denote the shapelet with *S* and the segment with *T*. Based on the above definition of *D*, we introduce δ by aligning the shapelet’s first data point with the segment’s first data point. When *S* and *T* are positioned like this, we can use *D* to measure the distance between *S* and the initial chunk (sub-segment) of *T* that is of the same length as *S* (we denote this chunk with *T’*). This will yield some value for the distance. Now we shift *S* along *T* by one data point (toward later time). *S* is now aligned with a different chunk of *T* (which overlaps with the previous chunk except in one point on the left and one on the right). We measure that distance and obtain a new value. We keep repeating this procedure: translate (shift) *S* along *T* point by point and measure the distance *D* at each step. We finish this when the end point of *S* aligns with the end point of *T*. The process is illustrated in Figure 4. We now define the distance δ between *S* and *T* simply as the minimal distance found during this shifting process (Xi et al., 2006; Ye and Keogh, 2009; Hills et al., 2013):

δ(S,T)=minT′[D(S,T)′].

Here, we denote with *T’* the consecutive chunks of the segment *T*, so that δ is the minimal distance *D* when all possible *T’* are considered. Thus defined δ meets the requirements for distance in the mathematical sense.

Note that this minimal distance is found when *S* is aligned with a specific chunk of *T*. In other words, the distance δ between *S* and *T* is actually the distance *D* between *S* and the chunk of *T* that is *most similar* to *S*. Therefore, there is a specific *best matching location* for *S* along *T*, at which it overlaps with chunk *T*’ to which it has minimal distance, as illustrated in Figure 4. So, when some *S* and some *T* are close, it means that *T* includes a chunk that is very similar to *S*. Note that the interpretation of δ also depends on the level of shapelet *S*. It is easier for δ to be small when *S* is short.

Now, for each member in the pool of shapelets we can measure the distance to all segments in the time series. Note that the distance between a shapelet and the segment to which it belongs is always zero, for all levels. Shapelets having small distances to other segments will be more “characteristic” for that time series. For example, shapelets belonging to sleep segments will typically have small distances to other sleep segments, since many HRV patterns recur during sleep. Similarly, “awake” segments will typically be similar to other awake segments. The relevance of these distances can be tuned by varying the resolution, i.e., changing the shapelet level. This property can be used to put together all segments belonging to sleep in one class and segments belonging to awake in another class, i.e., make classification of segments.

### Circadian (Diurnal) Patterns in Heart Rate Variability Data From Shapelet Distance Matrices

We next look at all-to-all distances between segments (for simplicity we call it distance between segments, even though by definition the distance is measured only between a shapelet and a segment). We create a matrix of distances between all pairs of segments as follows. We take a shapelet from the first segment of the measurement, and calculate the distance from that shapelet to all other segments in 24 h. These values fill up the first row in our matrix. Then, we take a shapelet from the second segment, and calculate the distances to all other segments in 24 h, filling up the second row in our matrix. Repeating this process, we arrive to the last segment and pick one of its shapelets, whose distances to all other segments fill up the last row in our matrix. Note that this is a square matrix and its size is the number of segments in 24 h. In our matrix, the element *i-j* reports the distance from a shapelet belonging to the *i*-th segment to the *j*-th segment (which we here confuse with the distance between *i*-th and *j*-th segment). Meanwhile, the element *j-i* will report the distance from a shapelet belonging to *j*-th segment to the *i*-th segment. Note that this matrix is not (necessarily) symmetric, since it depends on the choices of shapelets. However, in further analysis this matrix will be considered as symmetric, since our calculations indicate that this non-symmetry mismatches are negligible.

This setup depends on the choice of shapelet, specifically since we wish our distances to be interpretable as distances between pairs of segments. To this aim we consistently take an equivalently positioned shapelet in every segment. Specifically, we chose the last one in the first row of level 2 (cf. Figure 3). We tried several options for this analysis and this choice gave the most interpretable results (we do not report the entire choosing procedure).

**FIGURE 5:**
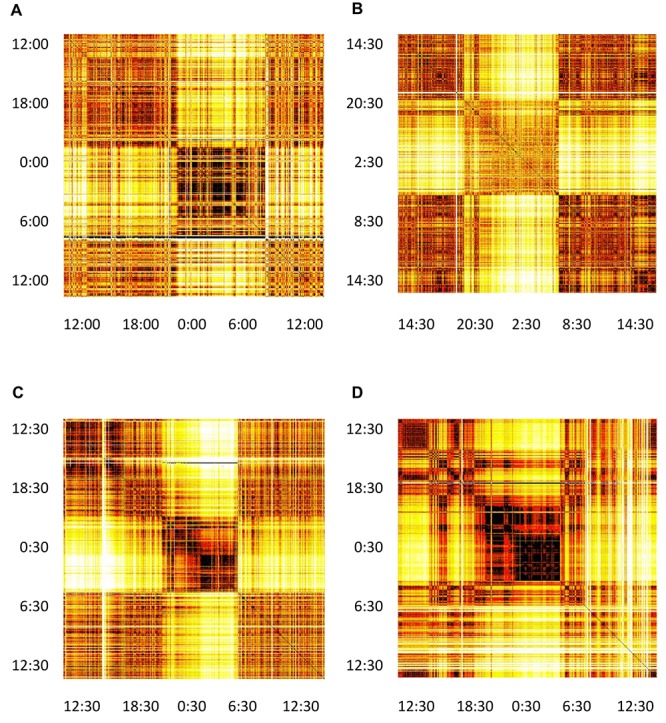
Distance matrices for four selected subjects visualized as heat-maps. **(A)** Male subject, 21 years of age, **(B)** Female subject, 23 years of age. **(C)** Male subject, 49 years of age. **(D)** Female subject, 50 years of age. All matrices have 24 h of measurements indicated both horizontally and vertically. The color of each matrix element indicates the distance between the two segments, identifies as *x* and *y* coordinate of that matrix element (we neglect the asymmetry of this matrix, see section “Discussion” in the text). Darker colors represent shorter distances and brighter colors larger distances, so that darker cells indicate that two segments have similar heart activity, while brighter colors connect segments with different heart activity. It can be noted that sleep shows more self-similarity than awake.

Proceeding with the computations as described above, we obtain a distance matrix for each subject. We represent it as a heat-map (color-map), where the color in each matrix element i-j indicates the distance between the i-th and j-th segment. In Figure 5 we show matrices for the same four typical subjects from Figure 2. All matrices offer a clear picture of sleep/awake difference: (almost) all sleep segments are similar to most other sleep segments (dark), but different from most awake segments (light), with corresponding comparisons obtaining for awake segments. The timing of falling asleep and waking up can also be identified for all four subjects and it agrees well with the self-reported information. Some qualitative patterns vary between younger subjects (top two panels) and middle-aged subjects (bottom panels). Of course, segments that are far apart have different heart activity, whereas those that are close have similar heart activity. In addition to the main sleep and awake stages, we see many short periods of opposite stage within both sleep and awake. For example, the person can relax or “take a nap” for a short period of time during the day, which is visible as different coloring within otherwise awake stage. Similarly, shallow sleep, arousals or even shortly waking up is seen in all panels. Interestingly, besides these intermittent changes of status, sleep state shows distinct patterns that might reflect various sleep phases (REM vs. deep sleep). This suggest the presence of short arousals/awakenings during sleep, possibly in relation to (Dvir et al., 2018).

## Determining The Onset Of Sleep Via Best Shapelet

We now extend the above analysis and construct a method to pinpoint precisely the onset of sleep using shapelet analysis. That amounts to finding in a time series the point (or points) during 24h where the time series (heart activity) qualitatively changes the most. We can safely claim that these points correspond to the changes of awake status. Namely, since the subjects observed their normal daily routines (refraining from sport and exercises) we do not expect these changes to reflect anything else. There are at least two such points in 24 h, one for falling asleep and one for waking up. However, as noted earlier with Figure 5, there could be more such points. In practice, we wish to classify all 2 min segments into two distinct groups based on the similarities of their time series patterns quantified via shapelet analysis. These two classes should (roughly) correspond to the parts of distance matrices colored differently. Note that this information on the onset of sleep will be *independent* from the self-reported information.

### Identifying the Best Split Point Between Sleep and Awake

**FIGURE 6:**
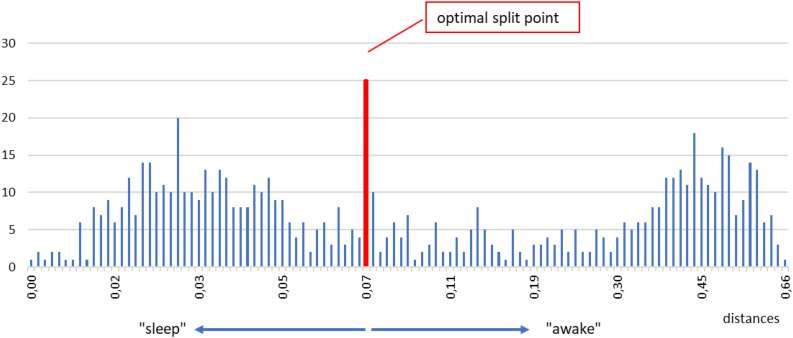
Example of a histogram of all distances between the selected shapelet (taken from sleep, just for illustration) and all segments in a time series. Two main peaks mentioned in the text are visible, one for smaller distances, one for larger distances (denoted as “sleep” and “awake” for easier orientation). To find the optimal split point we test all tentative split points between each two histogram bins. We do so by computing the information gain corresponding to each tentative split point and choose the one that gives the best (maximal) information gain. The optimal split point for the histogram above is marked by a vertical red line. Segments whose distances are smaller (respectively, larger) than the optimal split point are defined as belonging to the first (respectively, second) class. After repeating this procedure for the entire pool of shapelets we define the best shapelet as the one, whose optimal split point gives the maximal information gain. We call this best split point and consider it the output of our method for determining the onset of sleep.

Below we describe our procedure step by step. First, we take one shapelet, belonging to any segment and any level. We compute the distanceδfrom this shapelet to all segments in the time series. We put all those distances in a histogram. An example for a typical subject is shown in Figure 6. In such a histogram, small distances will cluster in one (or more) peaks near zero, whereas large distances will accumulate in other peak(s) away from zero. In fact, such grouping is seen in Figure 6, one peak around 0.03 and the other around 0.45. The reason for this grouping is clear: segments with short distances are chiefly those belonging to the same consciousness state as the chosen shapelet (sleep, for illustration), whereas segments with large distances are by and large belonging to the opposite state (awake, for illustration). What we want in our histogram, is that these two peaks are as separated as possible, so that the corresponding segments can be classified in two distinct groups as clearly as possible.

How to split the histogram into two optimally distinct parts? In formal terms we are looking for the *optimal split point*: the point on the horizontal axis at which the histogram can be most meaningfully divided into two parts. This is an optimization/classification problem that can be approached in several ways. We resort to information theory (MacKay, 2005; Cover and Thomas, 2006; Delgado-Bonal and Martín-Torres, 2016) and proceed as follows. We examine a tentative split point between two adjacent bins and compute the *information gain* (*IG*) for the corresponding division. *IG* quantifies how meaningful it was to split the histogram this way. To compute *IG* we label the two classes of segments with A and B. Segments with distances smaller than the split point belong to class A, and segments with larger distances to class B. To compute *IG*, we first define the *entropy E* of such a division as (Uğuz, 2011; Sonka et al., 2015; Shapiro, 2019):

$$E\,\left(D\right)=-p\,\left(A\right)\cdot \log_{2}\left(p\,\left(A\right)\right)-p\,\left(B\right)\cdot \log_{2}\left(p\,\left(B\right)\right).$$

Proportions of the segments in class *A* and B are *p(A)* and *p(B)*, respectively. We have *p(A)* + *p(B)* = *1*. That is to say, for each division of segments into two classes, we can define the entropy *E* of such a division via the above formula. Naturally, the above defined entropy is maximal when the segments are split in two equal classes, while it is minimal when all segments are in one class and none in the other class. Entropy will be used to determine information gain.

**FIGURE 7:**
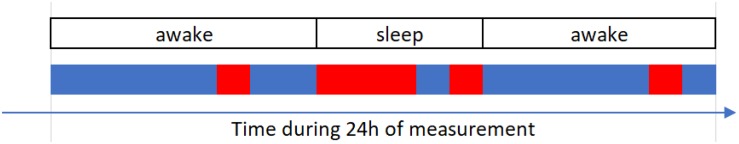
Conceptual (illustrative) representation of the classification of segments. Red and blue lines represent sleep and awake stages, respectively, obtained via our classification. Self-reported information is indicated in boxes. Actual sleep and awake stages overlap only partially with the stages as reported by the subject.

Before proceeding further, we recall that sleeping and awake segments are not homogeneous time-wise, since subjects can briefly change their consciousness status during “formal” sleep and awake. However, we are interested in distinguishing the sleep from awake states, regardless of when and in how many pieces it occurs. That is to say, “taking a nap” during the day is to be classified as sleep. A typical situation is illustrated in Figure 7.

Still, our focus is determining the onset of the real sleep, when the subject intentionally fell asleep. Namely, other changes of the awake status are not intentional and it is not clear how will they be reflected in the data. Hence, our next step is to improve the way we determine the optimal split point by including the self-reported information.

Considering a tentative split point, most segments classified as A belong to either self-reported sleep or self-reported awake stage. Let us assume for a moment that A belong to sleep. Then, most segments classified as B will belong to self-reported awake, but not all (see Figure 7). Similarly, there will be some segments classified as A, which will according to self-reported information belong to awake. We wish our optimal split point to account for this as best possible. We want to minimize the number of “misclassified” segments, or at least to have them as similar as possible to “correctly” classified segments of the same kind. In other words, we want to improve the information provided by the subject. We thus define *IG* starting with the general formula (Mitchell, 1997; Carmel et al., 2002; Ye and Keogh, 2009; Rakthanmanon and Keogh, 2013):

$$I\,G=E\,{\left(D\right)}_{b\,e\,f\,o\,r\,e}-E\,{\left(D\right)}_{a\,f\,t\,e\,r},$$

which states that *IG* is the difference in entropy before and after the splitting. More precisely, *IG*, as the difference of these two entropy values, is also weighted average entropy of both subsets after splitting, and can be expressed via formula:

$$I\,G=E\,\left(D\right)-{\frac{n_{a\,w\,a\,k\,e}}{n_{t\,o\,t\,a\,l}}}^{*}\,E\,\left(D_{a\,w\,a\,k\,e}\right)-{\frac{n_{s\,l\,e\,e\,p}}{n_{t\,o\,t\,a\,l}}}^{*}\,E\,\left(D_{s\,l\,e\,e\,p}\right).$$

Here, E(D) is calculated via previous formula, *n*_*total*_ is the total number of segments in 24h, *n*_*awake*_ and *n*_*sleep*_ are the total number of segments classified in the class where majority of segments, respectively, belong to awake and sleep according to self-reported information. Values *E(D_*awake*__)_* and *E(D_*sleep*_)* are obtained by considering the fact that “misclassified” segments represent a splitting of its own within self-reported sleep and awake stage. Hence, we calculate them using the earlier formula for Entropy, but now considering “correctly” classified vs. “misclassified” segments.

To sum up, for any tentative split point, *IG* quantifies how much are we better off considering that split point than self-reported split point. Then, the *optimal* split point is defined as the one for which the information gain is maximal. Such split point represents the best improvement of information obtained via splitting with respect to the self-reported information. With this in mind, we try each tentative split point, calculate the *IG* associated with it, and identify the split point leading to maximal *IG* as the *optimal split point*.

Furthermore, we note that the above procedure allows us to find the optimal split point for any shapelet in the pool of shapelets. Each optimal split point comes with its own *IG*. But these values of *IG* can be compared, and in particular, the maximum among them can be identified. We call it *best split point* and the shapelet corresponding to it the *best shapelet*. It is the shapelet whose optimal split point comes with the maximal *IG* compared to IGs associated with all other shapelets. Such shapelet provides a natural way to divide the original time series into two groups (classes) of segments with qualitatively distinct properties. It is in accordance with the best shapelet that we make the definite classification of segments into sleep and awake in what follows.

But before proceeding, we note that the above procedure could depend on the choice of bin size in our histogram. A histogram bin may be larger or smaller, so to test how appropriate was our choice, we employ the Levene’s test (Bland and Altman, 1996; Zimmerman, 2010; NIST, 2017). This test will assess the equality of variances for two groups with respect to bin size. The first group consists of 1%, 2%, 3% grades and the second 10%, 20%, 30% grades. Data in first group and in second group have different variance (*p*-value 0,029). Next we perform the Levene’s test and establish the difference in variance of group with histogram resolutions (1%, 2%, and 3%). In short, we found that the choice of bin size plays little or no role for our analysis. To close this description, we illustrate this entire procedure in a block diagram for easier orientation.

### Classification of Time Series via Best Shapelet

**FIGURE 8:**
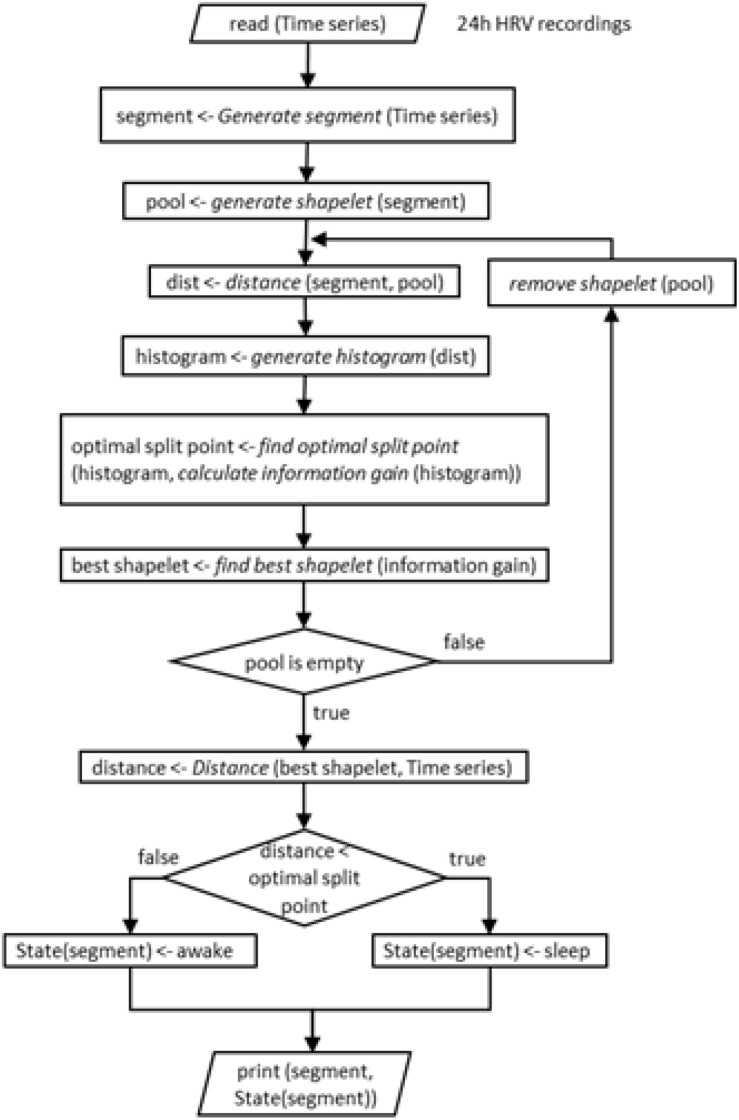
Schematic representation (block diagram) of the entire procedure of our shapelet analysis and identification of best shapelet. It is shown here for easier orientation. Illustration is again done for a shapelet selected from sleep.

**FIGURE 9:**
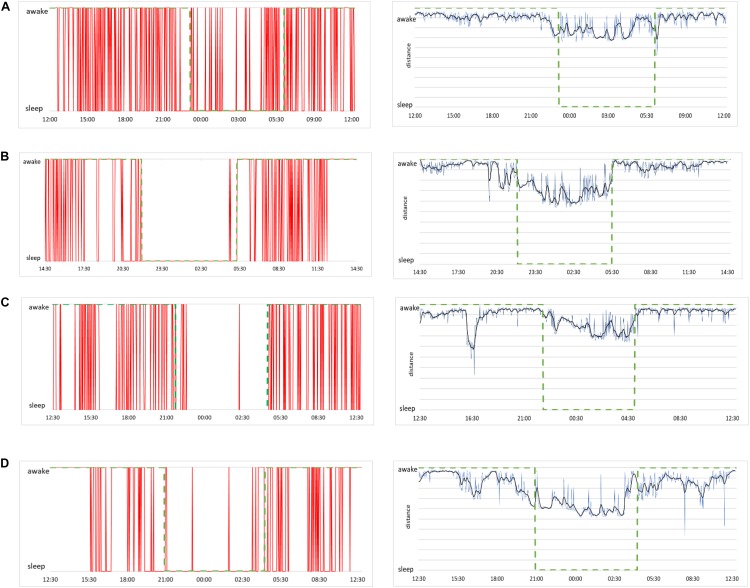
(**A**, left) Sleep and awake stages determined by our method for the case of a male subject, 21 years of age. Red lines indicate consciousness status as a binary value (sleep or awake) as a function of time over 24 h of measurement. (**A**, right) Raw data of distances from the best shapelet as function of time for the same subject (blue line), smoothed via Butterworth filter (black line) for better clarity. Green dashed line shows the self-reported information in both plots. **(B)** The same plots for a female subject, 23 years of age, **(C)** a male subject, 49 years of age, and **(D)** a female subject, 50 years of age.

To illustrate the classification into sleep and awake via best shapelets we consider the same four subjects as in Figure 2. Of course, computation on each of their HRV data leads to a different best shapelet, characteristic for their time series. We obtain the classification as described above explained in more details in Figure 8 and show the results in Figure 9 (left panels). Consciousness status determined by our approach is shown vertically (red line) as a function of time during 24 h. The green dashed line denotes the self-reported information. Our method correctly indicates that the subject is sleeping when (most likely) he/she is indeed sleeping, with some exception in Figure 9A, where the subject has had somewhat erratic sleep. In contrast, awake status is determined less precisely, since we see many intermittent intervals of sleep stage, which possibly account for subject relaxing or resting with reduces vigilance. Actually, this “conservatism” in establishing wakefulness is desirable in the context of, for example, traffic applications, where one needs an alarm system that goes off at the initial stage of fading vigilance.

To see how our method’s output aligns with shapelet distance, we show the distance from the best shapelet for the same four subjects in Figure 9 (right panels, blue line). Noisy profiles are smoothed for better clarity (black line). Indeed, the first subject seems to have had “shallow” sleep, as his heart activity seems less qualitatively different during sleep. This conclusion comes from observation that even the best shapelet was not discriminatory enough to establish a clear separation in distance statistics, which explains why the method found his sleep to be less stable. However, even in this case, our method identified many (potential) changes of awake status, even if some of them are identified incorrectly. Other subjects’ sleep was more stable, as correctly found by our method. This confirms that our method is made to indicate awake status only when that status is perfectly clear and that all intermediate stages of subject’s consciousness are defaulted as sleep.

### Pinpointing and Predicting the Onset of Sleep

**FIGURE 10:**
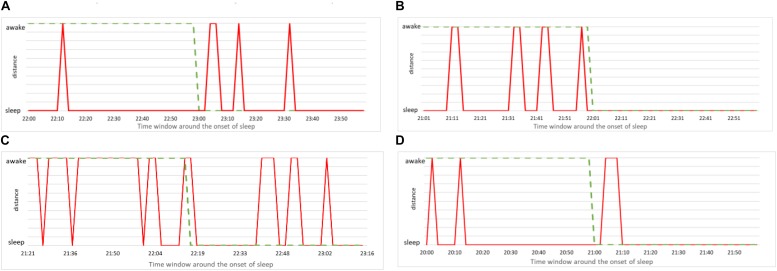
**(A)** Zoom to the onset of sleep for male subject, 21 years of age. Red line indicates the consciousness status determined by our method as a binary value (sleep or awake) within time window of 1 h prior and 1 h after the self-reported time of falling asleep. Green dashed line is the self-reported information. **(B)** The same for female subject, 23 years of age. **(C)** The same for male subject, 49 years of age. **(D)** The same for female subject, 50 years of age.

The simplest way to put our method to practical use is to make the alarm go off each time the above analysis indicates that the subject is asleep. Since our method robustly predicts wakefulness, we can reliably claim that a subject is indeed awake whenever our method indicates him/her to be awake. In formal terms this means we have many false positives – instances of the method indicating sleep while subject is (most probably) awake. Of course, from the practical viewpoint, false positives are more desirable than false negatives – instances when the method indicates wakefulness while the subject is asleep. Nevertheless, for our method to be of practical use, we need to examine how false positives can be reduced. To this aim we study more closely the performance of our method in the vicinity of the onset of sleep. We report again the data from Figure 9 but this time zooming to the time window of 2 h around the self-reported time of falling asleep (1 h before and 1 after). The results are shown in Figure 10, where we magnify the information from the panels on the left side of Figure 9, around the onset of sleep. Recall that this determination of awake status is independent from subject’s self-reported information. For the case (a), our method indicates that subject is classified asleep much before he reported to be asleep. While such a conservative determination is in principle desirable for practical purposes, this situation is a false positive that can hinder the operation of the alarm system. On the other hand, our method performs best in case (c), where the subject’s status comes out as awake almost entire actual awake time and as sleep almost immediately after the subject (most likely) fell asleep. Cases (b) and (d) are again showing the conservative performance of our method, indicating that subjects are asleep before they (most likely) were actually sleeping. This could be due to them relaxing for the bed time, which is reflected in their cardiorespiratory interaction that gradually becomes more “sleep-like.” Nevertheless, in the context of realistic applications of our method, excessive relaxation can lead to fading of vigilance so triggering an alarm in such a situation could be a good strategy.

**FIGURE 11:**
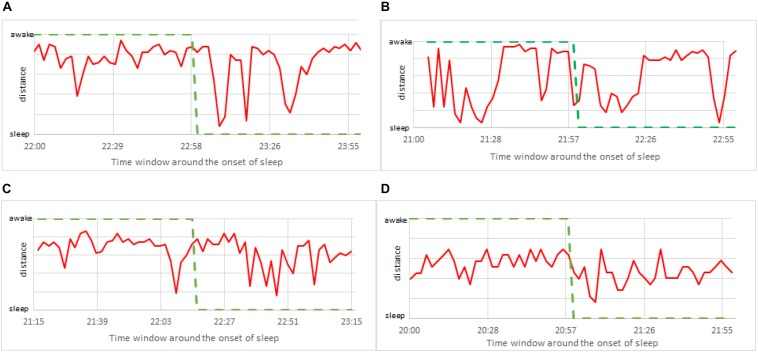
Zoom to the onset of sleep as in Figure 10, but this time calculated via 50 randomly chosen shapelets as described in the text (not via single best shapelet). The consciousness status is no longer binary (0 or 1), but a continuous value ranging between sleep (0) and awake (1). **(A)** Male subject, 21 years of age. **(B)** Female subject, 23 years of age. **(C)** Male subject, 49 years of age. **(D)** Female subject, 50 years of age.

To further improve the precision of detecting the exact moment of fading vigilance, we note that above results are obtained using *only one* shapelet. And even if this shapelet is the best shapelet, it is likely that classification via other shapelets will also contain useful information. Therefore, it makes sense to average the results obtained via several different shapelets (not necessarily best), expecting that each of them (depending on its size and position) will contribute additional information. To this aim we re-do the analysis from Figure 10, but now we average over 50 randomly chosen shapelets. Randomization is done not just via level, but we also introduce a random shift (not just half length as in Figure 3), and take shapelets from random location during 24 h. Averaging over all 50 thus obtained classifications, we obtain the results shown in Figure 11, where consciousness state is a continuous value ranging between 0 (sleep) and 1 (awake). Lines, colors and time window are as in Figure 10.

This insight clearly gives more flexibility in determining the consciousness status. For example, one could adjust the alarm to go off at a prescribed value between 0 and 1, when the vigilance level is deemed too low. Note that pure sleep and awake in these plots mean that almost all 50 shapelets indicate them as such, which offers a more stable classification. But still, confronting with self-reported information (green curve), the actual correlation is weak. This suggests that focusing on the timing of the onset of sleep might not be so useful for practical applications. Perhaps simply quantifying the vigilance might be a better defined and more useful problem to solve. We carried out the same analysis for all 75 subjects in our sample. Findings were similar to the four representative subjects in above two figures. Our method is systematically quick to signals sleep. What is also very clear, is that the heart activity at the onset of sleep strongly depends on the particularities of each individual. Universal trends that could be used to standardize our method are very hard to find.

**FIGURE 12:**
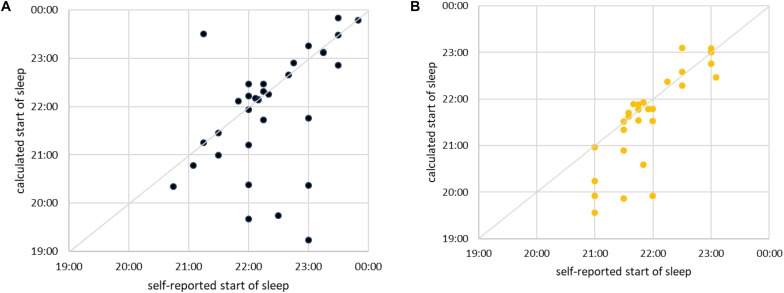
**(A)** Scatter plot of self-reported time of falling asleep (shown horizontally) vs. time of falling asleep calculated via best shapelet as described in the text (shown vertically) for male subjects. Each subject is shown as one point. The diagonal is marked for easier orientation. For simplicity and easier comparison, we took only subjects who fell asleep between 19:00 and 00:00. **(B)** The same for female subjects.

We next examine how good our method is in predicting (anticipating) the moment of a subject falling asleep. It is clear from the previous figure that this is hard, since at the onset of sleep our method indicates frequent transitions between sleep and awake. Yet for practical purposes the first such transition is important, since it suggests that the subject is definitely less vigilant, if not already asleep. Hence, we use our method to approximate the time of falling asleep as follows: We take the self-reported time of falling asleep and search for the nearest continuous interval composed of at least five consecutive segments of sleep (10 min). The beginning of such an interval is taken as the approximation for the time of falling asleep. If subject falls asleep exactly as he/she has indicated, the values will coincide. Now, we scatter plot the self-reported time of falling asleep against the time approximated as just described. The results for men are shown in Figure 12A and for women in Figure 12B. In most cases our method correctly identifies the transition (almost) immediately after it has occurred or even slightly before. However, in some cases our method is significantly too early or too late in determining the onset of sleep. As already discussed, that is due to large differences in sleeping transition from person to person. Our method (at this stage of development) is not sensitive to it. Yet, the fact that most subjects still lie along the diagonal is a promising sign. Our approach can, at least in principle, establish the onset of sleep for man and woman of any age and confirms that the increase of cardiorespiratory interaction starts before sleep occurs.

**FIGURE 13:**
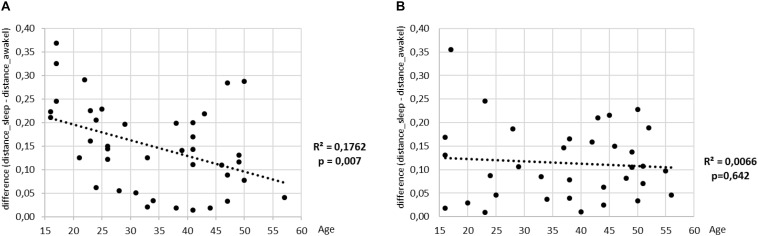
The value of mean distance from the best shapelet for one class minus the mean distance from the best shapelet for the other class (shown on *y*) is scatter plotted against the age (plots **A** for men and **B** for women). Regression lines are indicated in black, along with the respective values of *R*^2^ and *p*-value.

To finalize our analysis, we define a measure to quantify the discriminative power of our shapelet-based classification. To this end we consider again the histogram of distances from best shapelet (as shown earlier). Such histogram has two peaks, corresponding to two consciousness states, separated by the optimal split point. Now, for each subject we calculate the separation between those two peaks. Large separation means that heart activity is very different during sleep as opposed to awake, whereas small separation means the contrary. We scatter plot the values of this separation against the age for all subjects are show the results in (Figure 13). As expected, we find a good correlation for men. Interestingly, a negative correlation is significant for men, but not for women. It appears that the discriminatory power of heart activity to differentiate between sleep and awake decreases a lot more with age for men than for women.

## Discussion

We proposed a new method for automatically determining the consciousness status (sleep or awake) of a person from heart rate data only. Our method is based on shapelet analysis which looks for self-similarities in the data. By finding the best shapelet – the chunk of time series whose self-similarity properties allow for the best split of time series into two classes – we determine the awake and sleep states independently of information provided by the subjects, i.e., relying on HRV data only. The length of the best shapelet for most subjects is close to three lengths of a typical respiration cycle. This was somewhat expected, since RSA (synchronous modulation of heart by respiration rhythms) is known to be a reliable indicator of strong vagal activity and hence a reliable indicator of autonomic state (Moser et al., 2008). The method is developed to offer an individually optimal detection, but it can probably be extended to a longitudinal analysis of patterns, so that changes in behavior can be detected.

One of the applications of the approach is foreseen in public and general safety. Namely, our method can be developed into an alarm system that is triggered in case the system recognizes that the person under observation could be falling asleep. The paramount interest here is to have zero rate of false negatives. Our method seems to have covered this aspect rather well. In contrast, another goal is to minimize the number of false positives, but in this aspect, we encountered several limitations where there is room for improvement.

First, the proper way of calibrating our method would mean to have access to the precise timing of a subject falling asleep (“ground truth”). Note that while self-reported information is useful in narrowing our search, it is really not the ground truth: subjects can only report the time when the wanted to fall asleep, but not the time when they actually did. More complex experiments are needed to establish a reliable ground truth of the onset of sleep. A subject in a sleep laboratory could be simultaneously measured by Holter and by another device capable of independently determining the consciousness status. However, this will inevitably involve equipment that can disturb the sleep itself and the HRV measurements. Also, it is unlikely that any ground truth will be available in any practical (applicative) situation, so the ultimate interest are the methods that operate only with HRV data.

Second, further improvements of our method are possible through novel data analysis approaches. Specifically, note that in this work we used only the best shapelet to compute self-similarities. However, as a side result we found that splitting into just two classes of sleep and awake does not depend heavily on the choice of shapelet. In fact, many other shapelets have similar classification power. This means that one could cumulatively use several shapelets for classification, which would allow classification not as a binary value, but also to classify intermediate stages. Moreover, instead of 2 min segments, one can start with segments of shortened initial length and improve the resolution.

Third, another limitation of our method revolves around using prior data for individual subjects. In commercial applications this might be difficult, since the market might need an alarm system to work immediately and without prior data. This, however, is a very challenging task, especially due to particularities of each individual’s heart activity as he/she is falling asleep. On the other hand, having prior data for a period even longer than 24 h would allow for far more precise determination of consciousness status. In fact, this should also enable to predict the onset of sleep minutes ahead, rather than establishing the onset after it had happened.

Fourth, we realize that there are two different way of looking at the problem of determining the consciousness status. One way is to make determination for individuals only based on their prior data. Another is the search for universal patterns in everyone’s data and try to extract a universal method from those. Our work has shown that the first approach might be more promising in the short term. For any serious approach to the second approach one would need a far larger and more diverse sample of subjects. However, it is clear that the second way is more promising in terms of applications.

Fifth, we stress that we have focused on just one possible method of determining the onset of sleep from many conceivable methods. Clearly, a pressing issue revolves around comparing such methods and establishing which works best depending on the situation and the available information. Detailed comparison of these methods, while very important, is beyond the scope of this paper. But we note that such comparison might not be simple, since it will involve methods that operate on different foundations, for example, with or without ground truth.

Finally, our work has confirmed that shapelet analysis of cardiorespiratory interactions as present in HRV data is a useful tool. Except for methodological improvements mentioned above, this opens up further research questions. One of them has been mentioned already, namely, sleep phases could be studied via shapelet analysis. Shapelet distance matrices in Figure 5 reveal distinct patterns within sleep for all subjects, whose more detailed study is warranted.

## Data Availability Statement

The datasets generated for this study are available on request to the corresponding author.

## Ethics Statement

Ethical review and approval was not required for the study on human participants in accordance with the local legislation and institutional requirements. The patients/participants provided their written informed consent to participate in this study.

## Author Contributions

MM, AZ, and ZL envisaged the study. MF and MM arranged for the participation of subjects and collected the data. AZ designed and carried out the data analysis. AZ and MF designed the figures. ZL and AZ wrote, corrected, and carried the manuscript. All authors reviewed the manuscript.

## Conflict of Interest

The authors declare that the research was conducted in the absence of any commercial or financial relationships that could be construed as a potential conflict of interest.

## Footnotes

1 We confirm that we have obtained the permission from the copyright holder of this device to use the name of the device (ChronoCord) in this manuscript.

2 Choice of 2 min as the fundamental segment length is somewhat arbitrary. This choice involves two factors. Too short segments increase the computational cost, while too long segments deteriorate the resolution of analysis. By carrying out the shapelet analysis (described in rest of the manuscript) we found 2 min to be the best compromise.
